# Epigenetically regulated miR-1247 functions as a novel tumour suppressor via MYCBP2 in methylator colon cancers

**DOI:** 10.1038/s41416-018-0249-9

**Published:** 2018-10-15

**Authors:** Jennifer Liang, Wenchao Zhou, Nneha Sakre, Jennifer DeVecchio, Sylvain Ferrandon, Angela H. Ting, Shideng Bao, Ian Bissett, James Church, Matthew F. Kalady

**Affiliations:** 1 Department of Colorectal Surgery, Digestive Disease and Surgery Institute, Cleveland Clinic, Cleveland, OH USA; 2 Department of Stem Cell Biology and Regenerative Medicine, Lerner Research Institute, Cleveland Clinic, Cleveland, OH USA; 3 Genomic Medicine Institute, Lerner Research Institute, Cleveland Clinic, Cleveland, OH USA; 4 Department of Surgery, University of Auckland, Auckland, OH New Zealand; 5 Department of Cancer Biology, Lerner Research Institute, Cleveland Clinic, Cleveland, OH USA

## Abstract

**Background:**

Colorectal cancer (CRC) is a heterogeneous disease with distinct clinical subsets based on underlying genetic and epigenetic changes. DNA hypermethylation yields a unique CRC subset with a distinct phenotype and clinical behaviour, but this oncogenic pathway is not fully characterised. This study identifies and characterises miR-1247 as a novel tumour suppressor microRNA in methylated human colon cancers.

**Method:**

Tumour samples from patients with hypermethylated and non-methylated colon cancer and cell lines were evaluated for miR-1247 expression and function. A murine subcutaneous xenograft model was used for in vivo functional studies.

**Results:**

miR-1247 was methylated and underexpressed in methylator colon cancers. Overexpression of miR-1247 significantly inhibited cell proliferation, decreased tumour cell motility, induced apoptosis, and mitigated tumour formation capacity both in vivo and in vitro. Pharmacologic demethylation increased miR-1247 expression and produced similar anti-tumour activities. Mechanistic investigations revealed that MYCBP2, a member of the c-myc oncogene family, is a direct functional target of miR-1247. Furthermore, in CRC patients, MYCBP2 protein levels are associated with miR-1247 levels and survival.

**Conclusions:**

miR-1247 acts as a tumour suppressor by inhibiting MYCBP2 in methylator colon cancer. The MYCBP2/c-myc axis may underlie the anti-tumour activities of miR-1247 and is a potential therapeutic target via demethylation agents.

## Introduction

Colorectal cancer (CRC) is one of the leading causes of cancer-related morbidity and mortality worldwide^1^. It is well-established that multiple genetic and epigenetic alterations lead to the development of CRC with different clinical phenotypes and outcomes^2^. Two main oncogenic pathways, each with unique genetic and epigenetic patterns, have been described^3^: the chromosomal instability pathway (CIN) and the serrated or methylator pathway characterised by hypermethylation of DNA CpG islands (called the CpG island methylator phenotype, CIMP + ), with or without microsatellite instability. According to these criteria, CRCs can be broadly categorised as hypermethylated (CIMP + ) and non- methylated (CIMP-).^4–7^

The regulatory mechanisms that control the hypermethylated pathway have not yet been fully defined. However, epigenetic regulation of tumour suppressor genes contributes to cancer development.^8^ We have previously shown that hypermethylated CRC patients have worse clinical outcomes compared to non-methylated CRC patients^2^ and there is a need to further decipher these biologic and clinical differences.

MicroRNAs (miRNAs) are small non-coding, single stranded RNAs that regulate gene expression and influence many cellular processes such as proliferation, differentiation, and apoptosis. miRNAs function as tumour suppressors in various cancer types including CRC, and their expression can be regulated by DNA methylation.^9–11^ In depth analysis of previous work from our group has identified miR-1247 as one of only 2 differentially expressed microRNAs in hypermethylated CRCs with expression directly related to DNA methylation. In the current study, we have characterised its function as a novel tumour suppressor and identified MYCBP2 as its downstream target. Furthermore, we have demonstrated that manipulation of miR-1247 expression influences tumour growth and proliferation in vivo, thus opening the possibility for development of novel treatment options.

## Materials and methods

### Cell lines and clinical samples

The human colon cancer lines RKO and SW620 were supplied by Dr. Janet Houghton (Cancer Biology, Cleveland Clinic) and cultured in Dulbecco’s Modified Eagle’s Medium (DMEM) supplemented with 10% Fetal Bovine Serum (FBS). HCT116 and SW480 was purchased from ATCC and cultured in DMEM medium with 10% FBS. The Cleveland Clinic Department of Colorectal Surgery maintains an Institutional Review Board-approved protocol and the informed consent from each patient. Surgical samples have been characterised genetically by the presence of *KRAS* and *BRAF* mutations, microsatellite instability (MSI), and CpG island methylator phenotype (CIMP).^12^ Hypermethylated CRCs are characterised by *BRAF* mutations, CIMP+, and high microsatellite instability (MSI-H). Non-methylated CRCs are characterised by *KRAS* mutations, CIMP-, and microsatellite stability (MSS). Normal (non-adenomatous, non-cancer) colon tissues are also maintained in the biobank and were utilised for controls.

### Quantitative Real-Time PCR

Cells were harvested under exponential growth conditions. Quantitative Real-Time PCR (RT-qPCR) was performed to assess miR-1247 expression levels using TaqMan Universal PCR Master Mix (ABI 4324020). Briefly, miRNAs were isolated using the mirVana miRNA kit (Ambion AM1560) followed by reverse transcription with a TaqMan MicroRNA Reverse Transcription Kit (ABI 4366596). TaqMan PCRs were carried out with miR-1247-specific primers (ABI 4427975) or MAM6 control (ABI 4427975). PCR assays were performed using a 7900HT Sequence Detection System (Applied Biosystems). Samples were run in triplicate and standardised against endogenous MAM6 (Human Endogenous Control, Applied Biosystems). The resulting relative miR-1247 mRNA amounts in each sample were normalised to control values to yield fold changes.

### miRNA fluorescence in situ hybridisation

The formalin-fixed paraffin-embedded (FFPE) tissue slides were processed by using locked nucleic acid (LNA)–fluorescence in situ hybridisation (FISH) oligonucleotide probes for miR-1247 and miR-126 (Exiqon), both labeled with fluorescein at the 5′-end. We used miR-126 as a control for optimizing the probe conditions in these ISH experiments because it is expressed in colon tissue as instructed by the manual for the miRCURY LNA miRNA ISH Optimisation Kit 5 (*Exiqon*). The miRCURY LNA miRNA kit that we used has a double DIG-labeling detection probe that offers exceptional sensitivity and specificity to detect extremely low abundance miRNA. In order to detect the miR-1247 expression in methylator colon cancer we used miR-126 for optimizing the probe conditions as miR-1247 expresses at a low level in methylator colon cancer. After deparaffinisation, the slides were incubated with proteinase-K and endogenous peroxidase was blocked with 3% H_2_O_2_. Next, the slides were incubated with hybridisation mix containing 20 nM of doubled-DIG LNA miR-1247 probe in Hybridizer (DAKO Agilent, USA) for 1 h at 56 °C. The slides were incubated in blocking solution and antifluorescein–horseradish peroxidase antibody (1:125, PerkinElmer, Shelton, CT, USA) for 1 h. The signals were then amplified using Tyramide Signal Amplification for 15 min at room temperature. After incubation, the slides were mounted directly with Vectashield reagent and visualised with a fluorescent microscope (Leica). All steps beginning with hybridisation were performed in the dark.

### Genomic DNA isolation, bisulfite conversion, and methylation-specific PCR

RKO, HCT116, SW480 and SW620 cells were treated with 10 µM of 5-aza-2′-deoxycytidine (DAC) (Sigma-Aldrich, A3656) for 72 h. Genomic DNA (gDNA) was isolated using a QIAmp DNA Mini Kit (Qiagen, 51304). For methylation studies, 1 μg of gDNA was bisulfite-converted using the EZ DNA methylation kit (Zymo, D5001). Methylation-specific PCR was carried out using the EpiTect MSP kit (Qiagen, 59305) with methylation-specific primers (forward: 5′- GACAACGAAAAAACGTATCGAA-3′; reverse: 5′-TTTAGGTTTAGTGAGGAGTTTACGG-3′) or the nonmethylation-specific primers (forward: 5′-TTCAACAACAAAAAAACATATCAAA-3′; reverse: 5′-TTTAGGTTTAGTGAGGAGTTTATGG-3′) under the following conditions: 95 °C for 10 min; 94 °C for 15 s, 53 °C for 30 s, 72 °C for 30 s for a total of 40 cycles; 72 °C for 10 min. Universally methylated and non-methylated controls were purchased from Zymo Research (Zymo D5014). The PCR products were then analysed by 2% agarose gel electrophoresis.

### microRNA transfection

For transient transfections, 30 pM of miR mimic (Qiagen, 219600) or 30 pM inhibitor (Qiagen, 219300) were suspended in 6 μL of RNAimax (Life Technologies) to transfect 250,000 cells in a 35 mm dish according to the manufacturer’s instructions. Scrambled (All Star Negative) was also purchased from Qiagen. The cells were harvested and replated 48 h post-transfection for other experiments.

### Lentivirus package and infection

Lentiviral constructs containing miR-1247 (ABM Mh10072) were purchased from abmGood. For virus packaging, 293Ta cells were grown to 70% confluency and transfected with a lentiviral construct along with psPAX2 and pVSVG. At 48 h post transfection, the viral supernatant was collected and transduced into colon cancer cells at a multiplicity of infection (MOI) of 1. Transfection efficiency was monitored by green fluorescent protein (GFP) imaging. Stable expression of miR1247 was obtained by selecting infected cells with 1 μg/mL puromycin for 4 weeks until > 95% cells turned to being GFP-positive. The miR-1247 expression level in the stable cells was determined by qPCR.

### Cell proliferation, migration, and anoikis assays

For the cell proliferation assay, 500 cells/well were seeded in a 96-well plate 48 h after miRNA transfection. Cell viability was determined at indicated times with the CellTiter-Glo luminescent assay (G7571, Promega). Cell migration was examined in Transwell chambers (354578, BD Biosciences). 48 h post miRNA transfection, 50,000 cells were added into the upper well of each chamber, and migration assay was performed in DMEM or RPMI1640 medium with 10% fetal bovine serum for 24 h. Migrated cells were stained with 0.5% crystal violet and photographed with the microscope (Leica). Cell numbers were calculated with ImageJ. To assess cell resistance to anoikis, 50,000 cells were seeded in one well of a 24-well plate precoated with 10% poly-HEMA (P3932, Sigma) and cultured for 24 h. Cells were suspended in 0.4% Trypan blue (15250–061, Invitrogen) and viability was determined using a TC-10 automated cell counter (Bio-Rad).

### Tumour formation in nude mouse and demethylation treatment in vivo and in vitro

RKO cell line was plated at a concentration of 500 cells/well and 50,000 cells/well for the viability assay and miR-1247 RT-qPCR, respectively. The cells were allowed to attach overnight, and the next day were treated with either DMSO or 2.5 μM 5-aza-2′-deoxycytidine (DAC). 24 h later, the respective wells were transiently transfected with 30pM of an anti-sense miR-1247 inhibitor. Cells were harvested 48 h following transfection. Cell viability was determined at indicated times with the CellTiter-Glo luminescent assay. The levels of miR-1247 expression were analysed as previously mentioned using the miR-1247 TaqMan RT-qPCR assay.

HCT116 cells stably expressing miR-1247 or scrambled RNA were infected with luciferase-expressing lentivirus and 1 × 10^6^ cells were subcutaneously injected into the left or the right flank of athymic 5 week old C57BL/6 nude mice (Charles River Laboratory). Growth of the xenografts was monitored using an in vivo imaging system. One million HCT116 cells were subcutaneously injected into nude mice. In the treatment experiments, starting on the second day after injection, mice were treated with 5-aza-2′-deoxycytidine (decitabine, DAC) (A3656, Sigma-Aldrich) dissolved in dimethyl sulfoxide (DMSO) at 0.2 mg/5 g every day by intraperitoneal injection. The nude mice were maintained under specific pathogen-free conditions as approved by the Cleveland Clinic Foundation Institutional Animal Care and Use Committee (IACUC) and the experiments were conducted in accordance with the National Institutes of Health Guide for the Care and Use of Animals.

### Immunohistochemistry

Cryosections were incubated with the primary antibody overnight at 4 °C. The next day, sections were washed three times with phosphate buffered saline (PBS), followed by incubation with biotinylated goat-anti-rabbit secondary antibody diluted in PBS with 1% bovine serum albumin for 30 min at room temperature. Colour reaction of different sections was performed in parallel using a Vectastain DAB kit (Vector Laboratories) per the manufacturer’s instructions.

### Immunofluorescence

Cells grown on coverslips were fixed in 4% paraformaldehyde at room temperature for 15 min followed by permeabilisation in 0.3% Triton X-100 for 30 min. After blocking with 1% bovine serum albumin for 1 h, cells were stained with primary antibodies at 4 °C overnight. The next day, the coverslips were washed three times with PBS and incubated with Alexa-conjugated secondary antibodies along with Hoechst at room temperature for 1 h. Cells were mounted using mounting medium (Sigma) and visualised with a fluorescence microscope (Leica).

### Immunoblotting

Cells were scraped, washed with 1 × PBS and lysed in lysis buffer. Protein concentrations were estimated using Bradford reagent (Biorad). Equal amount of protein was loaded for immunoblotting. Following SDS-PAGE, resolved proteins were electroblotted on PVDF membrane (Biorad). The membrane was blocked overnight in PBS containing 0.1% Tween-20 (PBST) and 1 % BSA. The membrane was then probed with primary antibody in PBST for 2 h at RT or overnight at 4 °C followed by three 10 min PBST washes at room temperature. Incubation with the secondary antibody was done for 1 h, followed by three 10 min PBST washes prior to chemiluminescence detection using ECL substrate (Thermo Scientific). Following are the antibodies used for the immunoblotting: Anti-MYCBP2 polyclonal rabbit antibody (ab86078, Abcam), cleaved caspase-3 (9661 S, Cell Signalling Technology), c-myc (sc-40 Santa Cruz Biotechnology), and tubulin (T6074, Sigma-Aldrich) were used at a 1:200 dilution.

### 3′-UTR luciferase reporter assays

Plasmid containing full-length human *MYCBP2* 3′-UTR inserted downstream of the luciferase reporter gene was purchased from abmGood (MT-h15016). The luciferase reporter construct, along with β-galactosidase control plasmid, were transfected into 293 T cells. 24 h post transfection, the cells were split into 24-well plates followed by transfection of miR-1247 mimics or all-star negative scrambled mimics at 50 nM. At 72–96 h post miRNA transfection, luciferase activity was determined using a luciferase assay system (E4030, Promega) and normalised to β-galactosidase activity measured with a β-galactosidase enzyme assay system (E2000, Promega). The results represent three independent experiments, each performed in triplicate.

### Statistical analysis

Results for qPCR assays, cell viability, anoikis, and migration experiments were expressed as the mean ± SEM of at least three different experiments performed in triplicate. The data were analysed with GraphPad Prism (Intuitive Software for Science). Statistical significance was determined by *t*-test (two-tailed ungrouped) and the Mann–Whitney U test. All tests were two-sided, and a *p*-value of < 0.05 was considered statistically significant.

## Results

### miR-1247 expression is regulated by methylation in both colon cancer specimens and cell lines

**Fig. 1:**
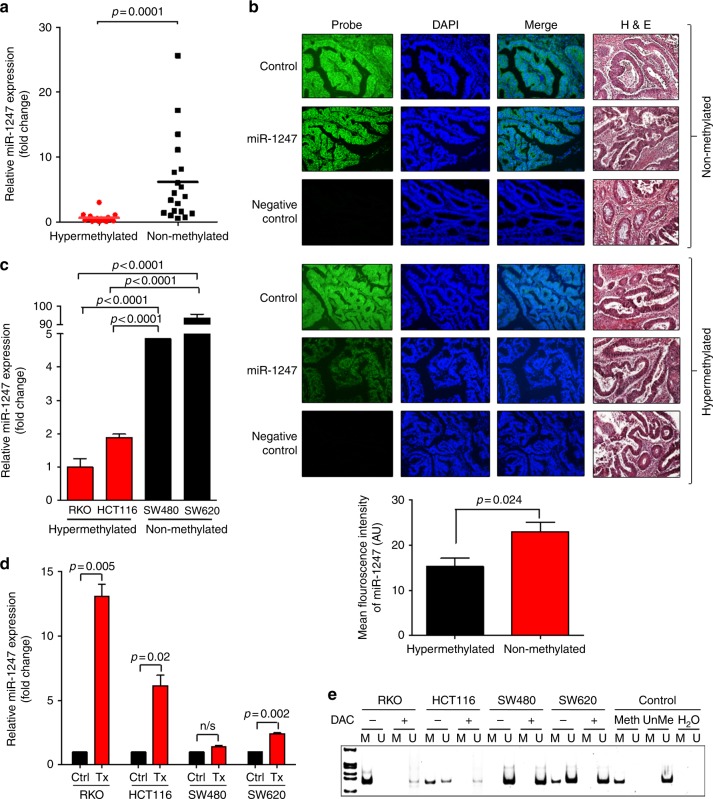
miR-1247 expression in hypermethylated colon cancer patient samples and cell lines. **a** TaqMan RT-qPCR from tumours of hypermethylated (*n* = 16) or non-methylated (*n* = 19) patients. Relative miR-1247 expression levels were calculated as fold changes by normalizing to normal colon samples (*n* = 10). **b** Fluorescence in situ hybridisation of miR-1247 on paraffin embedded tissues comparing hypermethylated colon cancer to non-methylated colon cancer. Control was realised by miR-126 probe, negative control corresponds to secondary antibody only. Representative images shown at ×20 magnification and quantifications of relative mean green fluorescence intensity of miR1247 in hypermethylated and non-methylated tissue samples was analyzed using ImageJ software. **c** TaqMan RT-qPCR of miR-1247 expression in hypermethylated vs. non-methylated colon cancer cell lines. Relative miR-1247 expression levels were calculated as fold changes by normalizing to Mock cells. **d** TaqMan RT-qPCR of miR-1247 expression levels in hypermethylated and non-methylated colon cancer cells after 10 µM 5-aza-2′-deoxycytidine (DAC) treatment during 72 h treatment. Results were calculated as fold change by normalizing to miR-1247 expression levels in each cell line treated (Tx) with DMSO (Ctrl). The fold change of the upregulated miR-1247 expression after the DAC treatment, was normalised to the baseline miR-1247 expression of respective cell lines. **e** Methylation specific PCR of *miR-1247* promoter region in hypermethylated and non-methylated CRC cells treated with 10 µM DAC during 72 h. M: methylation-specific primers. U: unmethylated specific primers

Promoter methylation plays an important role in regulating miR-1247 expression. Previously, we reported that miR-1247 is one of the most significantly methylated loci in colon cancer.^2^ Using the microarray data combined with genome wide methylation analysis, we found a distinct methylation pattern in the hypermethylated subtype of CRCs.^13^ To validate the relationship of miR-1247 expression to methylation status, we examined the miR-1247 expression levels in our colon cancer patient cohort using RT-qPCR and FISH (fluorescence in situ hybridisation) techniques in hypermethylated (*BRAF* mutant/*KRAS* wildtype/CIMP + /MSI-H) and non-methylated (*BRAF* wildtype/*KRAS* mutant/CIMP-/MSS) colon cancer subtypes (Fig. 1a). The expression of miR-1247 was analysed in hypermethylated (*n* = 16), non-methylated (*n* *=* 19), and corresponding normal tissue (*n* = 10) samples by RT-qPCR. The expression levels of miR-1247 were significantly lower in the hypermethylated CRC specimens than in the non-methylated specimens (*p* < 0.0001), (Fig. 1a). In agreement with the results of the RT-qPCR, in situ hybridisation results confirmed that the miR-1247 expression level was markedly reduced in hypermethylated CRC tissues (Fig. 1b, supplementary Figure S1). The quantifications of relative mean green fluorescence intensity of miR1247 in hypermethylated and non-methylated tissue samples was analysed using ImageJ software as shown in Fig. 1b (*p* = 0.024). Methylator and non-methylator colon cancer cell lines were chosen based on their genetic and methylator status (supplementary table, S1). Decreased expression of miR-1247 was also evident in hypermethylated CRC cell lines (RKO, HCT116) compared to the non-methylated cell lines (SW480, SW620) (Fig. 1c), confirming the applicability of these cell lines for use as an experimental model.

In order to identify the role of hypermethylation on miR-1247 expression, colon cancer cell lines were treated with the demethylation agent 5-aza-2′-deoxycytidine (DAC) or vehicle at 10 µM for 72 h. miR-1247 expression was determined by RT-qPCR. DAC exposure decreased mir1247 methylation levels, leading to an increase in miR-1247 expression in all cells, but the effect was less dramatic in non-methylator cell lines. Consistently, after DAC treatment, miR-1247 expression levels simultaneously increased in RKO ( > 10 fold) and HCT116 ( > 5 fold) cells, but only marginally increased in the non-methylator cell lines (Fig. 1d). Methylation-specific PCR (MSP) showed the hypermethylation in the *miR-1247* promoter region only in the hypermethylated cell lines (RKO and HCT116), but not in the non-methylated CRC cell lines (SW480 and SW620). DAC treatment reduced the methylation levels as shown in Fig. 1e. Taken together, the data demonstrates differential expression of miR-1247 based on methylation status of colon cancer subtypes.

### miR-1247 inhibits anchorage-independent survival and colon cancer cell line proliferation via cell apoptosis

**Fig. 2:**
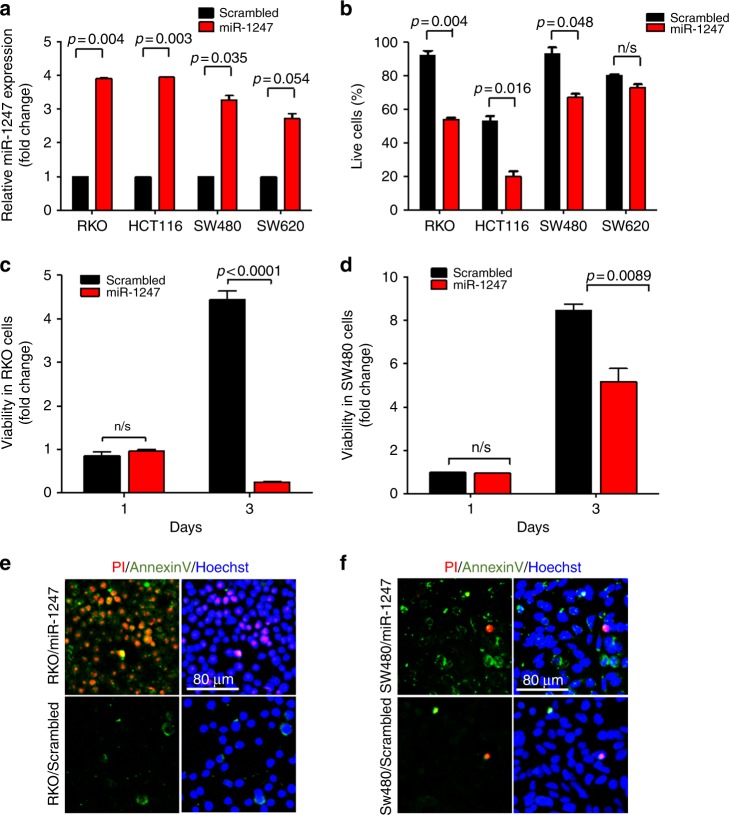
miR-1247 inhibits anchorage dependent cell survival and proliferation by promoting cell apoptosis in hypermethylated colon cancer. **a** TaqMan RT-qPCR of miR-1247 expression level after 48 h of miR-1247 mimic transfection. Results are represented as a relative fold change normalised to the baseline miR1247 expression of respective cell line. **b** Anoikis assay: viability of the cells was visualised after 24 h of miR1247 or scrambled transfection followed by trypan blue staining in a 24-well plate precoated with 10% poly-HEMA. **c** Cell proliferation of methylator RKO cells transfected with miR-1247 mimic or mock miRNA was estimated by cell titer. **d** Cell proliferation of non-methylated SW480 cells transfected with miR-1247 mimic or mock miRNA. **e**, **f** Annexin V (early apoptosis marker) plus PI (late apoptosis marker and necrosis) staining of hypermethylated RKO cell line or non-methylated SW480 cell line transfected with miR-1247 mimic or mock miRNA 3 days after transfection

The known rapid tumour progression of hypermethylated colon cancers^14,15^ prompted us to explore the role of miR-1247 in tumour cell survival and proliferation. Colon cancer lines were transiently transfected with either miR-1247 mimic or mock. After 48 h of transfection, miR-1247 expression levels were measured by TaqMan RT-qPCR and demonstrated a marked overexpression compared to respective scrambled-transfected cells (Fig. 2a).

Metastatic ability of cancer cells is often linked with resistance to anoikis, a form of programmed cell death that is induced by anchorage-dependent cells detaching from the surrounding extracellular matrix. The growth of miR-1247 mimic or scrambled miRNA transfected cells under anoikis conditions was investigated. Transfection of miR-1247 mimic significantly attenuated cell resistance to anoikis in RKO and HCT116 cells (Fig. 2b).

Overexpression of miR-1247 for 3 days resulted in a markedly impaired cell viability in the hypermethylated RKO cell line (Fig. 2c). Meanwhile, although cell viability of non-methylated SW480 cells was also impacted by miR-1247 overexpression, it was represented by a much smaller fold change between the scrambled and miR-1247 overexpressing cells when compared to the methylated cell line, RKO. (Fig. 2d). To determine the aetiology of the decreased viability, cell apoptosis was measured in the miR-transfected and mock-transfected cells by staining with Annexin V and propidium iodide (PI). The majority of RKO cells transfected with miR-1247 were Annexin V and PI double positive, indicating that the hypermethylated cells were at a late stage of apoptosis (Fig. 2e). Meanwhile, miR-1247 overexpression slightly increased Annexin V staining in SW480, suggesting a weaker apoptotic effect on the non-methylator cells (Fig. 2f). Similar results were observed in HCT116 and SW620 cells (supplementary Figures S2A and S2B). To further confirm that miR-1247 induced cell apoptosis, miR-1247 was overexpressed in HCT116 cells with lentiviral infection and apoptotic cells were detected with immunofluorescence staining of another apoptosis marker, cleaved-caspase 3 (Supplementary Figure S2C and S2D). Increased cleaved-caspase-3 positive cells were seen in miR-1247 infected cells compared to mock-infected cells (Supplementary Figure S2C). Taken together, these data show that miR-1247 suppressed colon cancer cell growth by inducing cell apoptosis and decreases cancer cell metastatic potential.

### miR-1247 regulates colon cancer cell motility

**Fig. 3:**
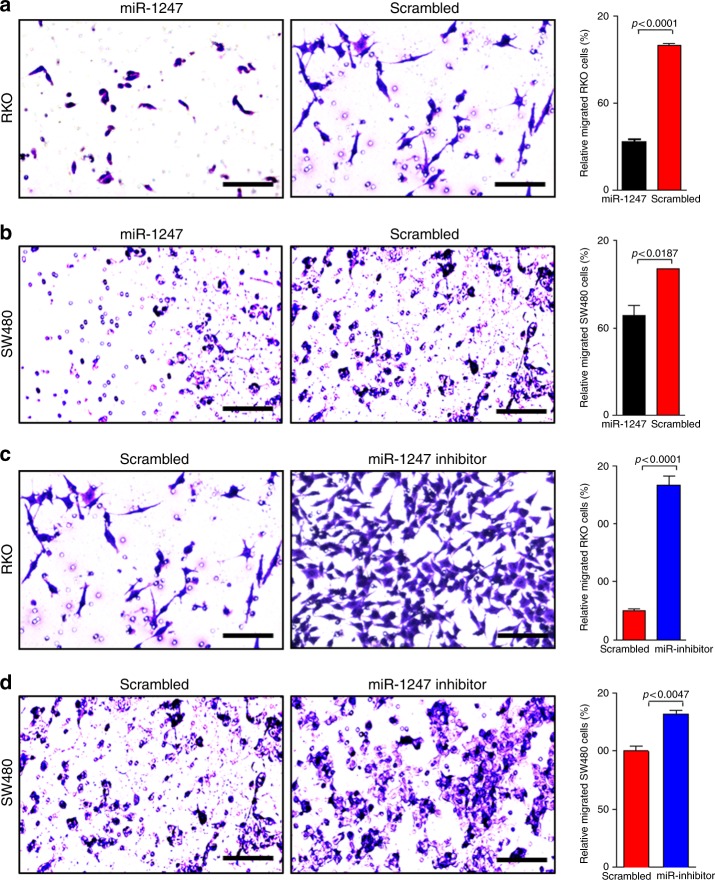
miR-1247 regulates colon cancer cell motility. **a** miR-1247 inhibits cancer cell migration in a transwell assay. The hypermethylated RKO cells were transfected with miR-1247 mimic or mock miRNA for 48 h before subjected to the transwell assay. The cells were seeded on a Matrigel precoated transwell membrane. The cells on the lower side of the chamber were stained, representing the number of cells that migrated. Cells treated with miR-1247 mimic exhibited less migration. Representative images are shown along with overall quantification. **b** Transwell assays of the non-methylated SW480 cells transfected with miR-1247 mimic or mock miRNA before subjected to the transwell assay and treatment and analysis are similar with transwell assay described above. **c** Transwell assays of the hypermethylated RKO cells transfected with anti-sense miR-1247 inhibitor or scrambled miRNA. After 48 h, cells (50,000) were subjected to transwell assay for 24 h. Cells transfected with the inhibitor demonstrated less migration. Representative images are shown along with overall quantification. **d** Transwell assays of the non-methylated SW480 cells was performed by transfecting cells with anti-sense miR-1247 inhibitor or scrambled miRNA. Histograms represent the quantification of the associated pictures in triplicates

Because cell motility is related to tumour progression and metastasis, we investigated the capacity of miR-1247 to regulate colon cancer cell motility.^16^ Cells transfected with miR-1247 mimic or scrambled miRNA were subjected to transwell assays. Overexpression of miR-1247 inhibited migration of both RKO and SW480 cells (Fig. 3a, b). The inhibitory effect was more significant in the hypermethylated (~ 70%) than that in the non-methylated cells (~ 30%) (Fig. 3a, b). To determine the role of endogenous miR-1247 on cell motility, cells were transfected with miR-1247 inhibitory anti-sense miRNA or scrambled miRNA and subjected to transwell assays. As expected, inhibition of endogenous miR-1247 significantly increased cell migration of hypermethylated cells (~ 400%) but only had a mild effect on non-methylated cells (~ 30%) (Fig. 3c, d). Overexpression of miR-1247 or its inhibitor in HCT116 and SW620 (Supplementary Figure S3A-D) cells recapitulated the results seen in RKO and SW480 cell line. Collectively, these data strongly suggest that miR-1247 can regulate colon cancer motility and migration.

### miR-1247 restoration via DNA demethylation by DAC regulates cell proliferation and migration in cell lines and xenografts

**Fig. 4:**
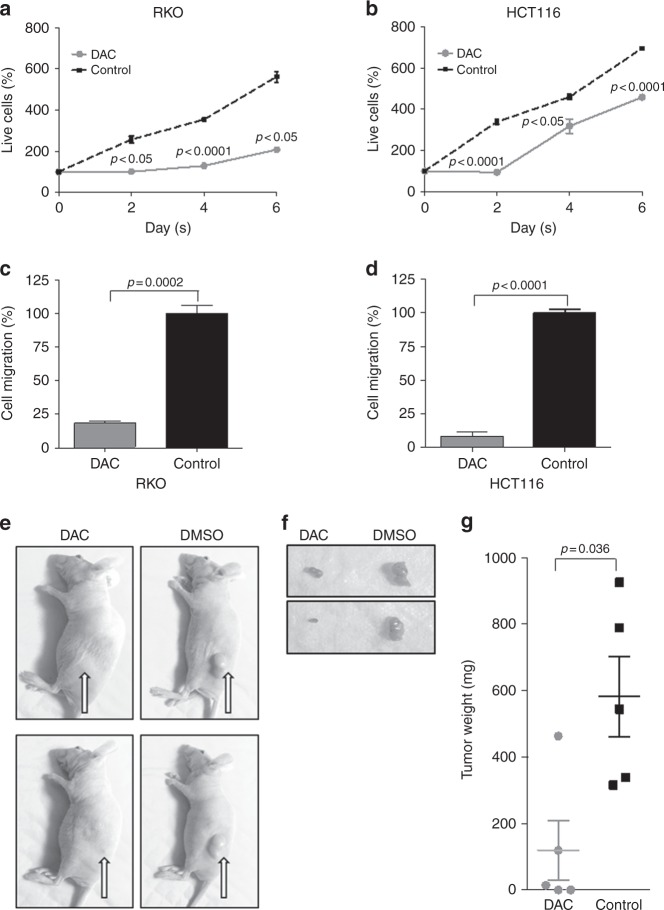
DAC treatment inhibits viability and motility of methylator colon cancer as well as suppresses colon cancer cell-derived xenograft growth. Viability curves of the hypermethylated colon cancer cells RKO (**a**) and HCT116 (**b**) treated with DAC or DMSO determined by cell titer. Cells were treated with DAC or DMSO and cell viability was monitored at the indicated time points. Transwell assays of RKO (**c**) and HCT116 (**d**) cells treated with DAC or DMSO. 50,000 cells were subjected to transwell assays for 24 h in corresponding media supplemented with 10% FBS. **e** Representative image of mice bearing HCT116 tumours treated with DAC (left) or DMSO (right). Photos demonstrated the tumour growth 30 days after injection. **f** Representative images of explants from DAC (left) or DMSO (right) treated mice. **g** Scatter plotting of the weight of explants with or without DAC treatment

To determine if DNA demethylation and subsequent increased miR-1247 expression translated into restored tumour suppression functions, colon cancer cells were treated with DAC and analysed by cell titer assays. Because methylation-specific PCR demonstrated that demethylation of the miR-1247 promoter region occurred in the hypermethylated cell lines (RKO and HCT116) but not in the non-methylator cell lines (SW480 and SW620) (Fig. 1f), the effect of DAC treatment on proliferation was analysed for hypermethylated cells only. Demethylation decreased cell growth of both RKO (Fig. 4a) and HCT116 cells (Fig. 4b). Furthermore, transwell assays of DAC-treated cells had decreased cell migration (Fig. 4c, d), which may represent a combined effect on cell growth and cell migration. To determine the relative contribution of miR-1247 restoration via demethylation, DAC-treated (2.5 µM) RKO and HCT116 cells were transiently transfected with miR-1247 inhibitor and proliferation was evaluated. In this particular experiment we have used lower concentration of DAC i.e. 2.5 µM to avoid the toxicity in the cells as we were looking at the effect of combination of two different treatments. Inhibition of miR-1247 after demethylation treatment resulted in approximately a 67 and a 25% decrease in cell proliferation in RKO and HCT cell lines respectively, vs. miR-1247- inhibitor alone. This suggests that miR-1247 is at least partly responsible for the effect seen after demethylation (Supplementary Figure 4A-D). For in vivo studies, one million HCT116 cells were subcutaneously injected into the flanks of nude mice. Mice bearing HCT116-tumours were treated with either DMSO or DAC once the tumours reached a volume of 200 mm^3^ (Fig. 4e). DAC treatment resulted in lower volume tumours, throughout treatment and persisted at 30 days after the injections (Fig. 4f). DMSO treatment yielded an average tumour weight of 582.4 mg, compared to an average tumour weight of 119.3 mg for DAC-treated tumours (Fig. 4g). Taken together, demethylation treatment of hypermethylated colon cancers increases expression of miR-1247 and restores tumour suppressor properties.

### miR-1247 suppresses colon cancer xenograft progression in vivo

**Fig. 5:**
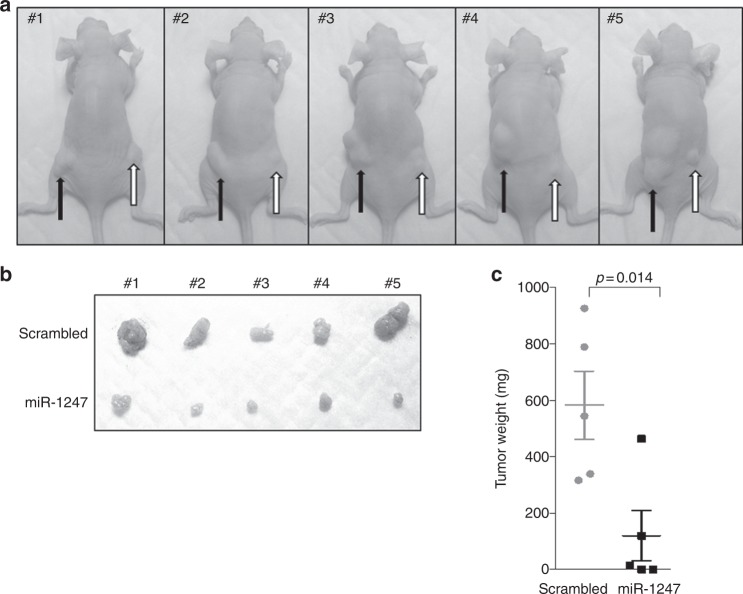
Overexpression of miR-1247 suppresses colon cancer cell-derived xenograft growth. **a** Image of mice bearing scrambled miRNA (black arrow) or miR-1247 mimic (white arrow) HCT116 cells xenografts at 30 days after injection (final time point). **b** Explanted tumours from scrambled miRNA (upper) or miR-1247 mimic (lower) HCT116 cells. **c** Scatter plot of the weight of the explants

To further define the influence of miR-1247 on colon cancer in vivo, we established a miR-1247 overexpressing HCT116 cell line tagged with EGFP (miR-1247 mimic-EGFP) and a control condition (scrambled). Stable miR-1247 expression in the infected cells was confirmed by EGFP reporter (Supplementary Figure S5A). Scrambled and miR-1247 expressing cells, were injected into the left and right flanks, respectively, of the same mouse. After 30 days, tumours established from miR-1247 overexpression HCT116 cells exhibited delayed progression in vivo (Fig. 5a). Furthermore, miR-1247 overexpression yielded significant reductions in tumour mass and size (Fig. 5b, c). Specifically, injection of miRNA scrambled cells or cells overexpressing miR-1247 cells yielded an average tumour weight of 424.5 mg and 73.3 mg, respectively, which represent a reduction of 82% in tumour mass (Fig. 5c). In studying the explanted tumours the vast majority of cells from miR-1247 xenografts had lost miR-1247 expression (Supplementary Fig. S5A and S5B). Before implantation of the cells, we confirmed the expression of the miR-1247 according to the expression of the EGFP reporter (Figure S5A). However, in the final xenografts, most cells were GFP- cells (Figure S5B). We believe this occurred because the forced expression of miR-1247 dramatically inhibited tumour cell growth and promoted cell apoptosis, as indicated in Fig. 2b and S2. Therefore, when growing in vivo in mice, the GFP + miR-1247 expressing cells proliferated at a very slow rate hence results in low or greatly lost the signal. Thus, reduction of miR-1247 expression delayed the tumour growth impart a survival advantage for these cells.

### miR-1247 targets MYCBP2 in colon cancer

**Fig. 6:**
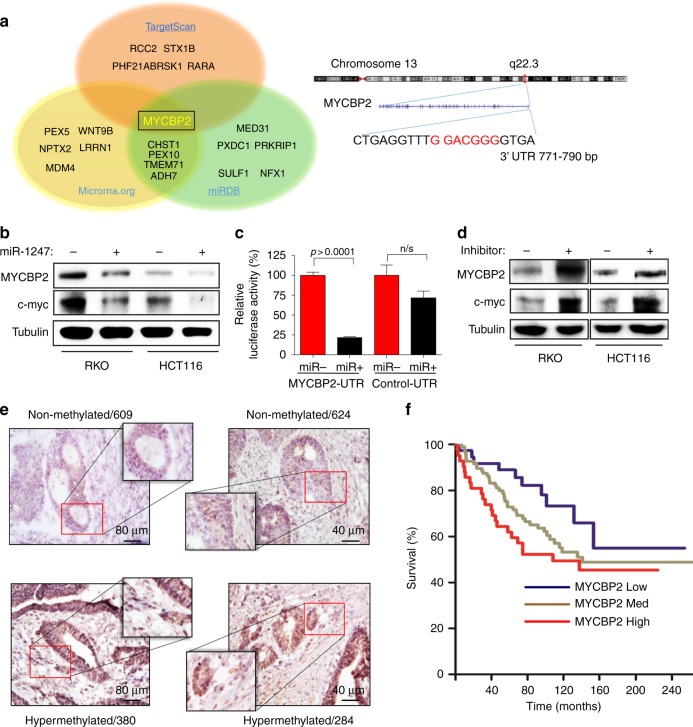
MYCBP2 is the direct target of miR-1247 in colon cancer. **a** Representation of predicted miR-1247 targets proposed by three predictive software programs TargetScan, microRNA.org, and miRDB. Overlap of the three different programs suggests myc binding protein 2 (MYCBP2) as a potential target. MYCBP2 binding location on chromosome 13. **b** Western blot of MYCBP2 and c-myc protein levels in hypermethylated cell lines overexpressing miR-1247 or scrambled RNA. miR-1247 overexpression reduced MYCBP2 and c-myc protein level 48 h after transfection. **c** Luciferase assays demonstrating miR-1247 targeting 3′UTR region of MYCBP2. **d** Western blot of MYCBP2 and c-myc protein levels in hypermethylated cell lines transfected with miR-1247 inhibitor or scrambled miRNA. **e** Representative immunohistochemistry of MYCBP2 staining in 2 hypermethylated colon cancers and 2 normal-methylated colon cancers. **f** Survival curve of the colon cancer patients with different MYCBP2 mRNA levels. Raw data were obtained from www.oncomine.com. Compared to the median MYCBP2 mRNA level of 177 patients, patients were classified as MYCBP2 low (MYCBP2 < −0.2, *n* = 38), MYCBP2 medium (−0.2 < MYCBP2 < 0.2, *n* = 97), or MYCBP2 high (MYCBP2 > 0.2, *n* = 42)

To predict the putative coding-gene targets of miR-1247, three predictive software programs TargetScan, microRNA.org, and miRDB were utilised. A common target proposed by these programs was myc binding protein 2 (MYCBP2) (Fig. 6a), a protein reported to bind to the well-known tumour associated transcriptional factor c-myc. To test this predicted target, the hypermethylated RKO and HCT116 cells were transfected with miR-1247 mimic or scrambled miRNA, lysed 48 h after transfection then immunoblotted for MYCBP2. As expected, MYCBP2 protein levels significantly decreased in miR-1247 overexpressing samples compared to scrambled overexpressing samples (Fig. 6b). Cells overexpressing miR-1247 also had decreased c-myc protein levels (Fig. 6b). To further clarify if the downregulation of MYCBP2 was specific to miR-1247 action, a MYCBP2 3′- UTR sequence containing binding site of miR-1247 was linked to a luciferase reporter gene. miR-1247 mimic and MYCBP2 3′-UTR luciferase constructs or control 3′-UTR (non-containing miR-1247 binding site) were co-transfected into 293 T cells. miR-1247 decreased luciferase activity by approximately 80% compared to scrambled (Fig. 6c). Meanwhile, miR-1247 overexpression had no significant effect on luciferase activity of the control construct (Fig. 6c). In addition, we studied the effect of miR-1247 inhibition on MYCBP2 protein level in RKO and HCT116 cell lines. Compared to control transfected cells, miR-1247 inhibitor transfected cells dramatically increased MYCBP2 protein level in hypermethylated cell line (Fig. 6d). Since the non-methylated CRC cells showed little responses to miR-1247 overexpression (Fig. 3b, d), we examined if MYCBP2 was regulated in a similar manner in this subtype of CRCs. Surprisingly, immunohistochemistry revealed that non-methylated cells exhibit trace amounts of endogenous MYCBP2, which could not be further suppressed by exogenous miR-1247 (Fig. 6e). Therefore, MYCBP2 is a miR-1247 target with predominance in methylator colon cancer cells. We further investigated if the inhibition of MYCBP2 expression by miR-1247 impacted the tumour associated transcriptional factor c-myc. Transfection of miR-1247 inhibitor elevated c-myc protein expression in parallel to the effects on MYCBP2 (Fig. 6d). Although the molecular mechanism of the positive regulation of c-myc by MYCBP2 remains unknown, these data demonstrate that miR-1247 directly targets MYCBP2, and is associated with decreased expression of c-myc, which may account for the tumour suppressive function of miR-1247.

Knowing that MYCBP2 expression is inversely correlated with miR-1247 in vitro, this relationship was then studied in five cryosections of hypermethylated and non-methylated patient colon cancers that were stained for MYCBP2 expression by immunohistochemistry. All of the hypermethylated samples demonstrated medium to higher MYCBP2 expression because hypermethylated samples have lower expression of miR-1247, whereas non-methylated samples showed no to low MYCBP2 staining, as higher expression of miR-1247 functions as a suppressor of MYCBP2 expression, (Supplementary Figure S6A and 6B). Collectively, these data suggest that MYCBP2 is a miR-1247 target and the miR-1247/MYCBP2/c-myc axis may serve as a tumour suppressive pathway in methylator colon cancers.

To explore the clinical relevance of MYCBP2 in CRC prognosis, we analysed a public mRNA microarray dataset, www.oncomine.com.^17^ One-hundred-seventy-seven patients were classified as MYCBP2 low (MYCBP2 < −0.2, *n* = 38), MYCBP2 medium (−0.2 < MYCBP2 < 0.2, *n* = 97), or MYCBP2 high (MYCBP2 > 0.2, *n* = 42) using the median MYCBP2 mRNA level for the entire population. Higher MYCBP2 mRNA levels correlated with a worse survival in CRC patients (Fig. 6f), which underscores the potential relevance of miR-1247 as a tumour suppressor with effects on MYCBP2.

## Discussion

The genetic and epigenetic mechanisms underlying hypermethylated colon cancers continue to be elucidated. Recent studies suggest miRNA dysregulation secondary to DNA hypermethylation may be a crucial step in CRC progression.^18–20^ Our previous work has demonstrated unique genome-wide CpG island methylation patterns between normal colon, non-methylated colon adenocarcinoma, and hypermethylated adenocarcinoma.^13^ Looking specifically at hypermethylated DNA regions associated with miRNAs, the promoter of miR-1247 was differentially methylated and expressed between hypermethylated and non-methylated colon cancers. Thus, we focused on miR-1247 expression as a potential driving force in hypermethylated CRCs. We have now demonstrated that miR-1247 is a novel tumour suppressor in hypermethylated colon cancers and that is regulated by epigenetic changes and is a target for therapeutic intervention.

miRNAs related to cancer initiation and progression are suppressed by DNA hypermethylation in several cancers.^21–23^ Such suppression influences tumour progression, metastasis, epithelial-to-mesenchymal transition, and resistance to 5-fluorouracil.^22,24–27^ Studies of DNA methylation-regulated miRNA have mainly focused on differences between normal and cancer tissues, but our work is the first to explore this concept in specific subtypes of colon cancers. Besides the differential expression of miR-1247 in hypermethylated vs. non-methylated cells, the effects of miR-1247 manipulation differed by subtype. For instance, although endogenous miR-1247 has a relatively lower expression in hypermethylated colon cancer cells, when it was inhibited by synthetic anti-sense RNA, the effects on cell motility were much greater in methylated cells than in non-methylated cells. This highlights the importance of miR-1247 as a tumour suppressor in the hypermethylated subset of colorectal cancers in terms of tumourigenesis and potentially in response to therapy. This has particular clinical implications in microsatellite unstable tumours, of which the vast majority are hypermethylated and do not respond to standard 5-fluoruracil based chemotherapy regimens.^28,29^ In addition to our findings in colon cancer, miR-1247 has recently been shown to acts as a tumour suppressor in in vitro models for pancreatic cancer and hepatocellular carcinoma.^30,31^ Thus, miR-1247 may have broader implications to cancer, but the downstream targets vary in these publications and the relationship may be tissue and context specific.^32^

Colon cancers arising via the hypermethylated pathway have a worse prognosis^12^ and may be less responsive to standard chemotherapy regimens. Therefore, alternative therapeutic approaches are needed.^28^ Epigenetically acquired changes to DNA are reversible, making them targets for therapeutic intervention. Azacitidine (5-azacytidine) is a United States Food and Drug Administration (FDA)-approved drug for the treatment of myelodysplastic syndrome with a favourable safety profile. Our study has shown that 5-aza-2′-deoxycytidine treatment at clinically relevant doses demethylates the miR-1247 promoter and increases expression of miR-1247, which decreases colon cancer cell viability, proliferation, and migration.^33,34^ This preclinical work supports miR-1247 as a target through which demethylating agents could provide a potential personalised adjunctive treatment specifically for the subset of methylator colon cancers.

Another novel discovery is the identification of MYCBP2 as a coding-gene target of miR-1247 for colon cancer. MYCBP2, also known as PAM (protein associated with myc), is an adenylyl cyclase and an E3 ubiquitin ligase that is a highly conserved protein that interacts directly with the transcriptional activating domain of myc.^35,36^ One study has reported MYCBP2 as potential target for miR-1247 in a prostate cancer model^37^ but not validated in clinical human tissues as has been shown in the current study. Previous studies in T-cell lymphoma showed a link between poor prognosis and MYCBP2 overexpression, indicating the involvement of MYCBP2 in cancer.^38^ Using in silico analysis of publicly available mRNA microarray datasets, we found higher MYCBP2 mRNA levels correlate with worse survival in CRC (Fig. 6f). Thereby, it is possible that miR-1247 exerts its tumour suppressive function by targeting the putative oncogene *MYCBP2*. The molecular mechanisms downstream of MYCBP2 are largely unknown. A striking discovery is the sharp decline of *c-myc* proto-oncogene expression in cells overexpressing miR-1247. Through regulating approximately 15% of all genes, *myc* proto-oncogene has global effects on cellular proliferation, differentiation, and apoptosis,^39–41^ and hence is considered a key oncogenic transcription factor. Although, MYCBP2 was initially identified as a myc-associated protein, no report has interpreted its direct interaction with c-myc. More studies are required to elucidate the mechanisms of *MYCBP2/myc* axis in tumourigenesis.

In addition to broader demethylating agents, specific miRNA expression can be manipulated as a treatment approach. Although still in its infancy, overexpression of miRNAs can be induced using either synthetic miRNA mimics or chemically modified oligonucleotides.^41^ Conversely, miRNAs can be silenced by antisense oligonucleotides and antagomirs.^42^ Although organ-specific delivery of miRNA remains a major challenge, luminal delivery of small RNA for the prevention of CRC in mice through use of bioengineered probiotic bacteria has been reported, and a similar strategy can be devised for miRNA.^43^ These studies suggest an exciting potential for miRNAs as a novel class of therapeutic targets and as a powerful intervention tool in CRC treatment.

In summary, we identify miR-1247 as a novel tumour suppressor that is epigenetically regulated in hypermethylated colon cancers. DNA hypermethylation plays a crucial role in this subtype of colon cancer by suppressing miR-1247 expression, which results in an increase in MYCBP2 and its downstream c-myc protein, thereby leading to tumour development. Use of demethylating agents (already in phase 1–2 trials for other diseases) and miR-1247 mimics may present a novel therapeutic approach to the treatment of hypermethylated CRC.

## Electronic supplementary material

Legends for Supplementary Figs and Table

S Fig 1

S Fig 2

S Fig 3

S Fig 4

S Fig 5

S Fig 6

Supplemetnal Table 1

Additional Information
