# Supplementary material for: Epigenetically regulated miR-1247 functions as a novel tumour suppressor via MYCBP2 in methylator colon cancers
Source: Br J Cancer. 2018 Oct 15;119(10):1267–77. doi: 10.1038/s41416-018-0249-9 (PMC6251029; doi:10.1038/s41416-018-0249-9)
Supplement: Supplementary file 1 — Legends for Supplementary Figs and Table [file 41416_2018_249_MOESM1_ESM.docx]

**Figure S1:** Fluorescence *in situ* hybridization of miR-1247 on paraffin embedded tissues comparing hypermethylated CRC to non-methylated CRC. miR-126 probe was used as control to optimize the conditions, negative control correspond to secondary antibody only. Representative image are shown with 20X magnification.

**Figure S2:** Annexin V (early apoptosis marker) plus PI (late apoptosis marker and necrosis) staining of A. hypermethylated HCT116 cell line or B. non-methylated SW620 cell line. C. miR-1247 was overexpressed in HCT116 cells with lentiviral infection and apoptotic cells were detected with immunofluorescence staining of another apoptosis marker, cleaved-caspase 3. D. Increased cleaved-caspase-3 positive cells were seen by immunofluorescence in miR-1247 infected cells compared to scrambled cells.

**Figure S3:** miR-1247 regulates colon cancer cell motility. A. The hypermethylated RKO cells were transfected with miR-1247 mimic or scrambled miRNA for 48 hours before subjected to the transwell assay. The cells were seeded on a Matrigel precoated transwell membrane. The cells on the lower side of the chamber were stained with crystal violet and the representative images are shown. B. Transwell assays of the hypermethylated HCT116 cells transfected with anti-sense miR-1247 inhibitor or scrambled miRNA. After 48 hours, cells (50,000) were subjected to transwell assay for 24 hours. Histograms represent the quantification of the associated picture.

**Figure S4:** miR-1247 restoration via DNA demethylation by DAC contributes to the decreased cell viability. Hypermethylated colon cancer cell lines RKO (A. B.) and HCT116 (C. D) were plated and pre-treated with 2.5µM DAC 24 hours prior to a transient transfection of an anti-sense miR-1247 inhibitor. A. and C. TaqMan RT-qPCR of miR-1247 expression level in both cell lines following 48 hours of anti-sense miR-1247 inhibitor transfection. Results are represented as a relative fold change of miR-1247 expression normalized by the control treated condition. B. and D. Viability of both RKO and HCT116 cell lines determined by Cell Titer Glo following 48 hours of anti-sense miR-1247 inhibitor transfection. Results are represented as a relative fold change of viability normalized by the control treated condition.

**Figure S5:** A. miR-1247 overexpressing HCT116-derived cell line tagged with EGFP (miR-1247 mimic-EGFP) and its corresponding scrambled observed by immunofluorescence. B. Stable miR-1247 expression was verified by EGFP reporter in xenografts.

**Figure S6:** A. Representative immunohistochemistry staining of MYCBP2 in two non-methylated colon cancers and, B. methylated colon cancers.

**Table S1:** Genetic and methylator status of different colon cancer cell lines.
