# Supplementary figures and images for: Epigenetically regulated miR-1247 functions as a novel tumour suppressor via MYCBP2 in methylator colon cancers
Source: Br J Cancer. 2018 Oct 15;119(10):1267–77. doi: 10.1038/s41416-018-0249-9 (PMC6251029; doi:10.1038/s41416-018-0249-9)

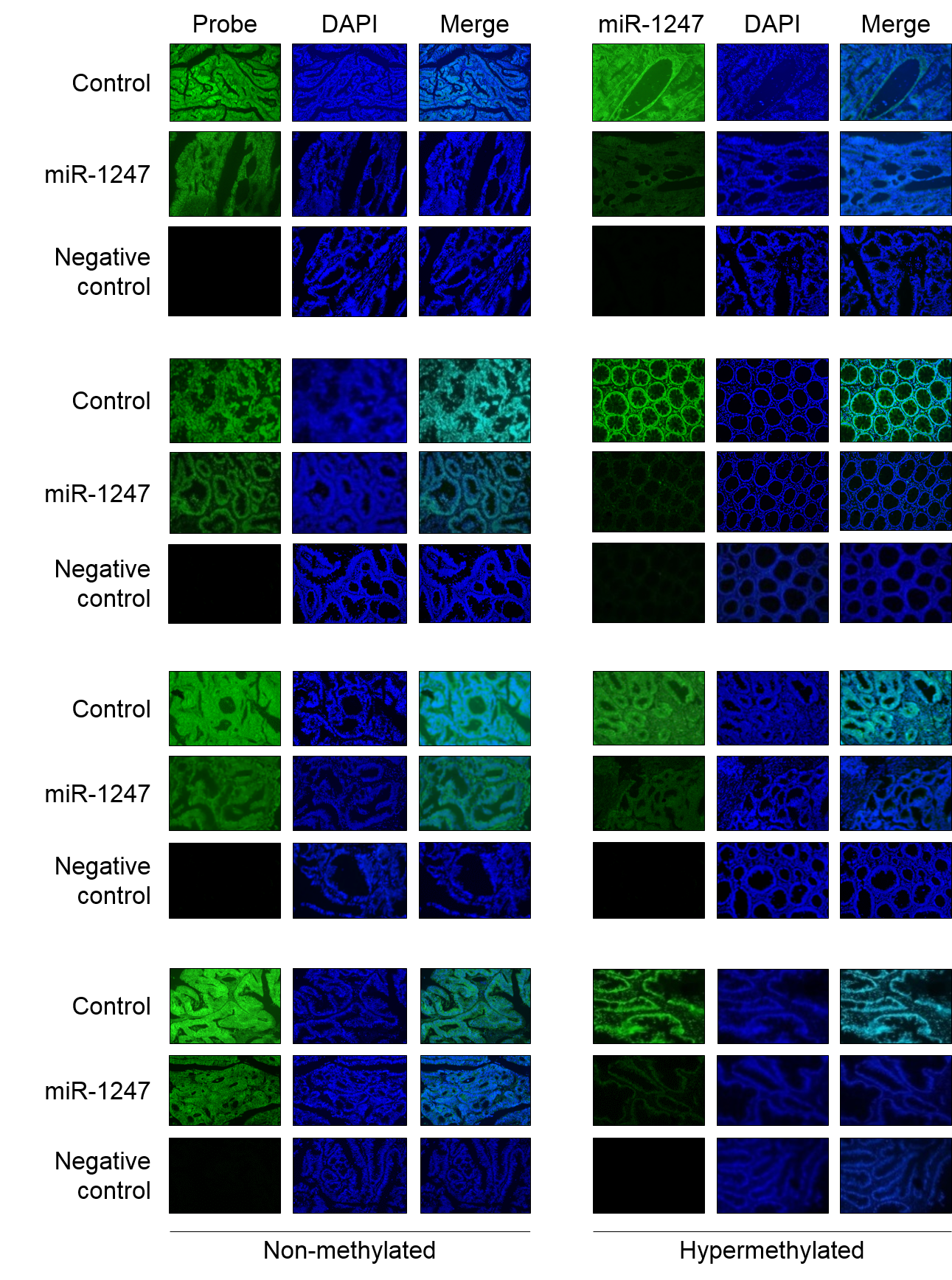

Supplement: Supplementary file 2 — S Fig 1 [file 41416_2018_249_MOESM2_ESM.tif]

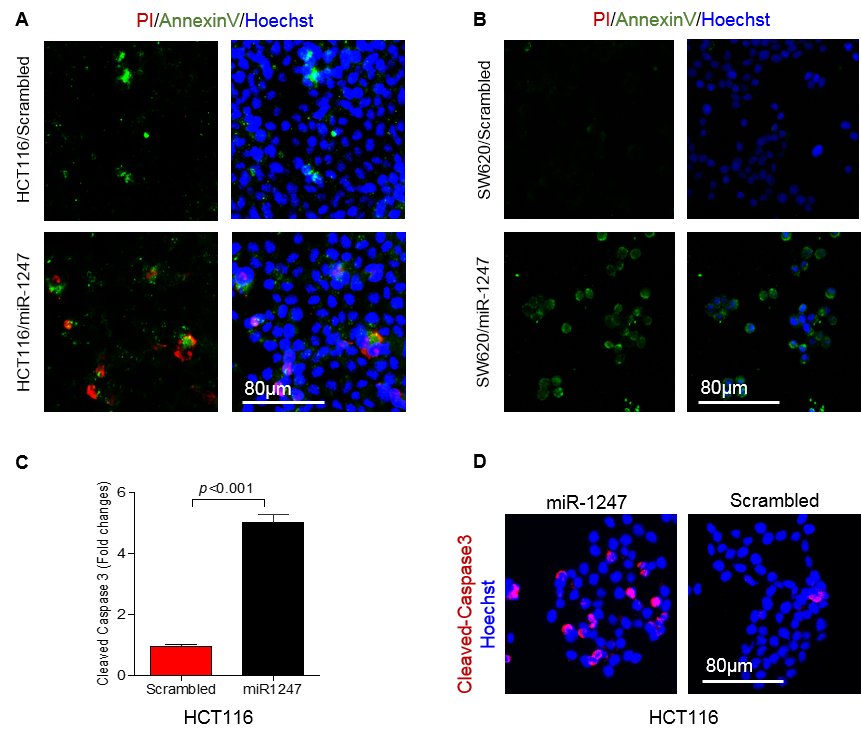

Supplement: Supplementary file 3 — S Fig 2 [file 41416_2018_249_MOESM3_ESM.tif]

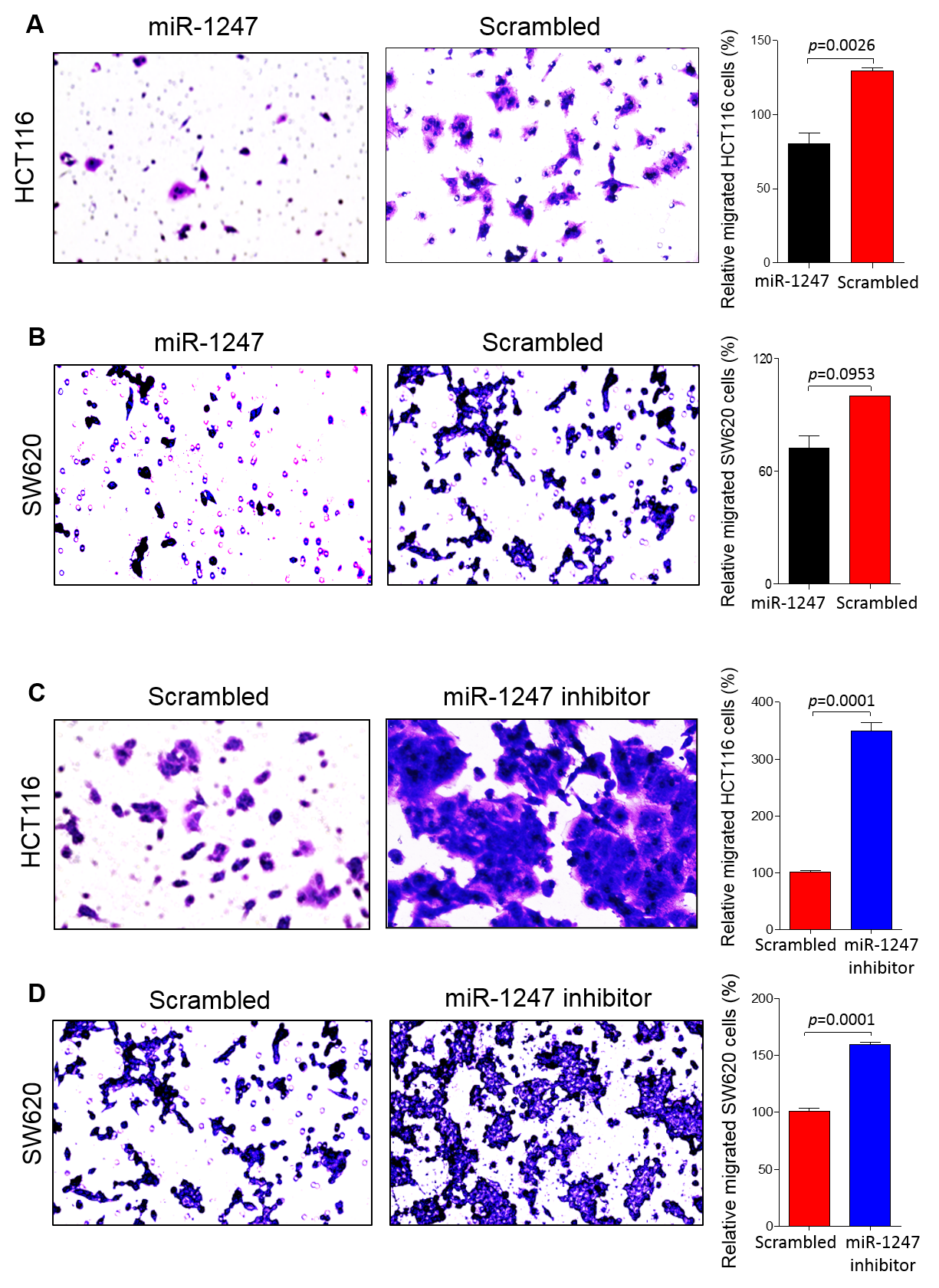

Supplement: Supplementary file 4 — S Fig 3 [file 41416_2018_249_MOESM4_ESM.tif]

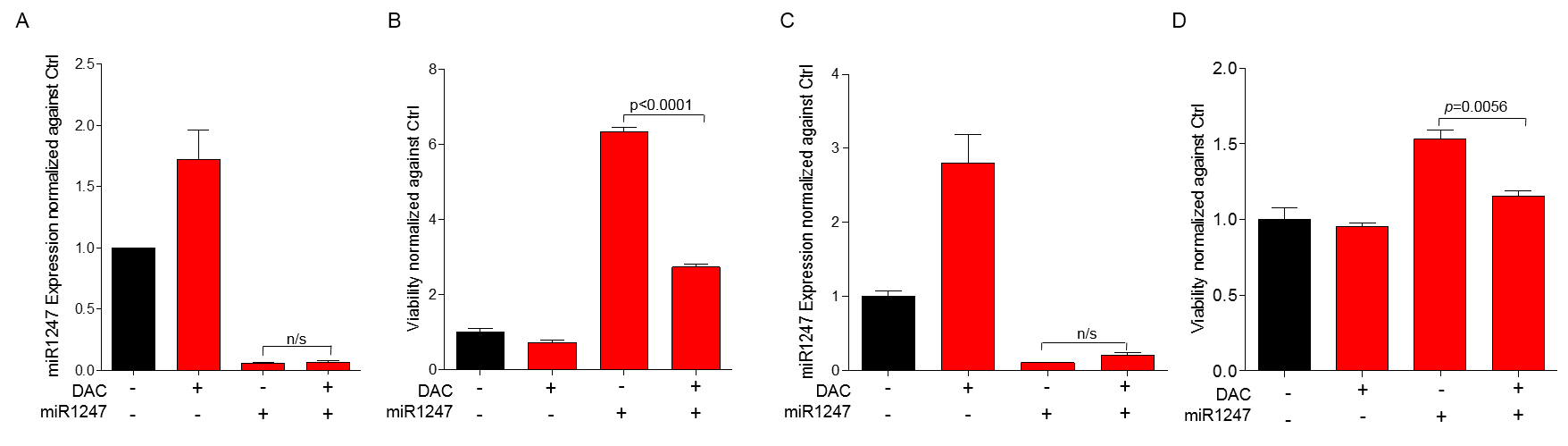

Supplement: Supplementary file 5 — S Fig 4 [file 41416_2018_249_MOESM5_ESM.tif]

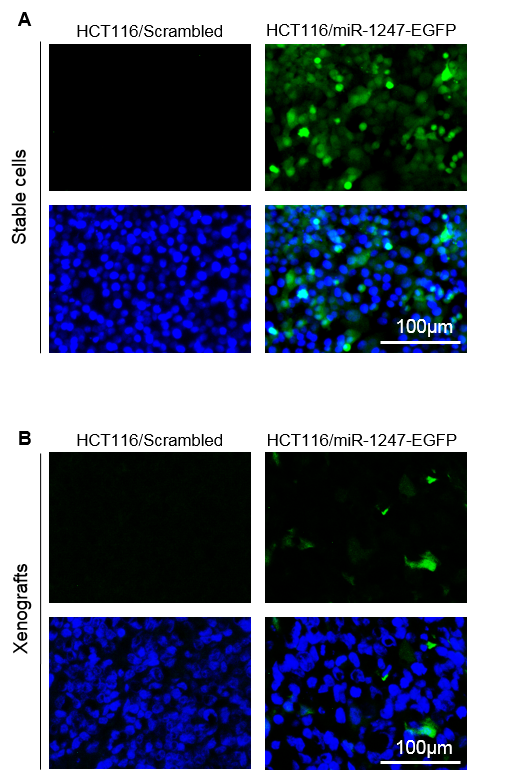

Supplement: Supplementary file 6 — S Fig 5 [file 41416_2018_249_MOESM6_ESM.tif]

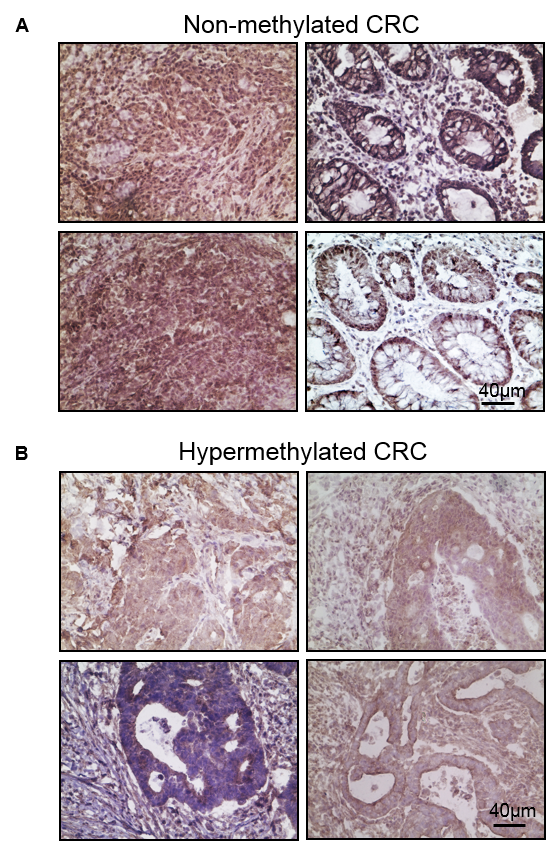

Supplement: Supplementary file 7 — S Fig 6 [file 41416_2018_249_MOESM7_ESM.tif]
