# Supplementary material for: Epigenetically regulated miR-1247 functions as a novel tumour suppressor via MYCBP2 in methylator colon cancers
Source: Br J Cancer. 2018 Oct 15;119(10):1267–77. doi: 10.1038/s41416-018-0249-9 (PMC6251029; doi:10.1038/s41416-018-0249-9)
Supplement: Supplementary file 8 — Supplemetnal Table 1 [file 41416_2018_249_MOESM8_ESM.docx]

| **Cell lines** | **Hypermethylated** | **MSI** | ***BRAF*** | ***KRAS*** |
| --- | --- | --- | --- | --- |
| SW480 | No | MSS | WT | Mutated |
| SW620 | No | MSS | WT | Mutated |
| RKO | Yes | MSI-H | Mutated | WT |
| HCT116 | Yes | MSI-H | Mutated | WT |
