# Supplementary material for: Epigenetically regulated miR-1247 functions as a novel tumour suppressor via MYCBP2 in methylator colon cancers
Source: Br J Cancer. 2018 Oct 15;119(10):1267–77. doi: 10.1038/s41416-018-0249-9 (PMC6251029; doi:10.1038/s41416-018-0249-9)
Supplement: Supplementary file 9 — Additional Information [file 41416_2018_249_MOESM9_ESM.docx]

**Additional Information**

*Ethics Approval:* This study is not a clinical trial. Tissue specimens that were included in this study were obtained within a Cleveland Clinic Institutional Review Board-approved studies (IRB# 4134 and #4192) biobank. Patients were consented as part of this biobank for allowance of tissues to be used for basic science research studies. As such, this study is waived for formal ethics approval. The Cleveland Clinic Foundation's Federal-wide Assurance, FWA00005367, has been approved by the Office for Human Research Protections (OHRP) and expires 5/14/2020. The Cleveland Clinic Foundation's IRB Organization Registration Information, IORG0000301, was updated 6/11/2018. The Institutional Review Board of The Cleveland Clinic Foundation is duly constituted (fulfilling FDA requirements for diversity), allows only those IRB members who are independent of the investigator and the sponsor of the trial to vote on the trial, has written procedures for initial and continuing review of clinical trials, prepares written minutes of convened meetings, and retains records pertaining to the review and approval process; all in compliance with requirements defined in 21 CFR Parts 50, 56, 312 and 812 and ICH (International Conference on Harmonization) guidelines relating to GCPs (Good Clinical Practice) that mirror FDA regulations. This study was performed in accordance with the Declaration of Helsinki.

All animal studies described were approved by the Cleveland Clinic Institutional Animal Care and Use Committee (IACUC) and conducted in accordance with the National Institutes of Health *Guide for the Care and Use of Laboratory Animals*. All studies were performed in the Biological Resources Unit (BRU) at the Lerner Research Institute as part of the Cleveland Clinic Lerner College of Medicine. The OLAW assurance number is A3047-01 (2018-2021). The services provided by the BRU encompass all aspects related to animal husbandry and experimental manipulation, ordering and procurement of animals, quarantine procedures, preoperative animal care, health surveillance and consultation on animal models. The Attending Veterinarian directs the Veterinary Services programs with the support of an Assistant Director and veterinary technicians. Approximately 40 animal technicians and husbandry staff, some of them certified by the American Association for Laboratory Animal Science (AALAS), provide daily animal care. Care is in compliance with the Animal Welfare Act and Public Health Services policies, and is conducted in conjunction with the Institutional Animal Care and Use Committee (IACUC). Cleveland Clinic animal care and use program was evaluated by the Association for the Assessment and Accreditation of Laboratory Animal Care, International (AAALAC) and received full-accreditation in March of 2008. The BRU’s facilities have been AAALAC accredited since 1976. All mice were housed in the BRU animal facility with HEPA-filtered and temperature, humidity, and lighting controlled. Mice, food, and water were also inspected daily by the laboratory’s technician. Cages and bedding are changed at least weekly. Any changes in behavior or sign of the following humane endpoints below will lead to early termination of experiments and immediate euthanasia of animals: 1) Behavioral changes (aggression, guarding, hiding); 2) Licking, biting, scratching, or shaking of procedure site or any other self-mutilation; 3) Changes in hair coat (ruffled fur, lack of grooming, piloerection); 4) Changes in posture or ambulation (tense, stiff gait, ataxia); 5) Non-weight bearing for 24hrs (difficulty walking, inability to maintain upright posture); 6) Changes in activity level (restlessness, pacing, lethargy); 7) Infection unrelated to the protocol; or 8) Signs of moderate to severe pain or distress which was not anticipated by the study plan. Buprenorphine, an analgesic administered s.c. (0.1mg/kg), will be administered as needed following treatment for pain management.

Consent for Publication: This manuscript does not contain an individual’s personal data that would require consent for publication.

Availability of Data and Materials: miRNA microarray data referenced in the manuscript can be found on [www.oncomine.com](http://www.oncomine.com). Any additional primary data will be available upon request.

Conflict of Interest: The authors have no competing interests related to the work in this manuscript.

Funding: The work for this manuscript was supported by the Krause-Lieberman Endowed Chair in Colorectal Surgery (MFK). MFK is supported by the NIH 1R01CA193359-01.

Authors’ Contributions: The authors contributed to the manuscript in the following ways:

**JL:** Conception and design, Collection and/or assembly of data, Data analysis and interpretation, Manuscript writing. **WZ:** Experimental design, Collection and/or assembly of data, Data analysis and interpretation. **NS:** Collection and/or assembly of data, Data analysis and interpretation,of experimental design, manuscript revision **JD:**  Collection and/or assembly of data, Data analysis and interpretation, Conception and design, revision of manuscript **SF:** Collection and/or assembly of data, Data analysis and interpretation, revision of manuscript. **AT:** Conception and design, Data analysis and interpretation, revision of manuscript. **SB:** Conception and design, interpretation of data, revision of manuscript. **IB:** Conception and design, interpretation of data, revision of manuscript. **JC:** Provision of study material or patients, data analysis and interpretation, Provision of study material or patients, Manuscript revision. **MFK:** Conception and design, administrative support, financial support, provision of study material or patients, data analysis and interpretation, Provision of study material or patients, manuscript writing and revision.

Acknowledgements: We would like to thank Dr. Ranjan Dutta and Christina Volsko of the Department of Neurosciences (Lerner Research Institute, Cleveland Clinic) for their technical guidance and reagents for the miRNA fluorescence *in situ* hybridization assay. We would also like to thank Hanumant Chouhan, M.D., for his valuable insights in manuscript preparation.
